# Heat stress induced apoptosis is triggered by transcription-independent p53, Ca^2+^ dyshomeostasis and the subsequent Bax mitochondrial translocation

**DOI:** 10.1038/srep11497

**Published:** 2015-06-24

**Authors:** Z. T. Gu, L. Li, F. WU, P. Zhao, H. Yang, Y. S. Liu, Y. Geng, M. Zhao, L. Su

**Affiliations:** 1 Department of intensive care unit, The Third Affiliated Hospital of Southern Medical University, Guangzhou, P.R. China; Department of Pathophysiology, Southern Medical University, Guangzhou 510515, P.R. China; 2 Southern Medical University, Guangzhou, P.R. China; 3 Department of Gerontology, 421 Hospital of PLA, P.R. China; 4 Department of intensive care unit, General Hospital of Guangzhou Military Command, Key Laboratory of Tropical Zone Trauma treatment and Tissue Repair of PLA, Guangzhou, PR China; 5 Department of Pathophysiology, Southern Medical University, Guangzhou 510515, P.R. China

## Abstract

In this study, We demonstrated that Bax mitochondrial translocation plays a vital role in the initiation of the mitochondrial signaling pathway upon activation by heat stress. In addition, both p53 mitochondrial translocation and Ca^2+^ signal mediated MPTP opening activate Bax mitochondrial translocation. Employing pifithrin-α (a p53 mitochondrial translocation inhibitor) and CsA (a permeability transition pore (MPTP) inhibitor), we found that heat stress induced Bax mitochondrial translocation was significantly inhibited in cells pretreated with both PFT and CsA. Furthermore, we demonstrated that generation of reactive oxygen species (ROS) is a critical mediator in heat stress induced apoptosis and that the antioxidant MnTBAP significantly decreased heat stress induced p53 mitochondrial translocation and Ca^2+^ signal mediated MPTP opening, as well as the subsequent Bax mitochondrial translocation and activation of the caspase cascade. Taken together, our results indicate that heat stress induces apoptosis through the mitochondrial pathway with ROS dependent mitochondrial p53 translocation and Ca^2+^ dyshomeostasis, and the ensuing intro Bax mitochondrial translocation as the upstream events involved in triggering the apoptotic process observed upon cellular exposure to heat stress.

Excessive heat gain from elevated ambient temperature has been proposed as a risk factor for the severely life-threatening disorder known as heat stroke, which is characterized by a rapid increase in core body temperature to above 40 °C, and accompanied by central nervous system dysfunction. Previous studies using cell lines and animal models have suggested that endothelial cells are an early target of heat stress injury and that damaged endothelial cells are prominent features of severe heat stroke^1^,^2^. Recent studies also demonstrate that endothelial cells can be induced to undergo significant apoptosis during the acute phase response to heat stress^3^. Although endothelial cell apoptosis appears to play an important role in heat stroke, the molecular mechanism by which heat stress induces endothelial-cell apoptosis is still poorly understood.

It is possible that the cytotoxicity of heat stress is mediated, in part, by oxidative stress^4^,^5^,^6^ and intracellular Ca^2+^ overload, or both^7^. These two factors are connected to each other. Oxidative stress induces calcium dyshomeostasis, and subsequently induces oxidative stress^8^. Furthermore, mitochondria are not only the a storage compartment for intracellular calcium, a source of oxygen radicals and a sensor for oxidative stress^9^,^10^, they also play a vital role in the regulation of apoptosis in response to a variety of death signal inducers including heat stress^11^,^12^. However, the chronology of cellular events that initiates calcium dyshomeostasis and increase in oxidative stress, which ultimately lead to the endothelial cell death observed in heat stress condition remains unknown. It has been well established that intracellular Ca^2+^ overload can induce cytotoxicity and trigger apoptotic cell death through activating two pathways, the mitochondria dependent and independent pathways^13^. The mitochondria dependent apoptotic pathway involves multiple events such as generation of ROS, loss of mitochondrial membrane potential, release of cytochrome c from mitochondria, opening of permeability transition pores, expression of the Bcl-2 family members and activation of caspases-9 and -3^14^,^15^. The mitochondria independent apoptotic pathway involves calpain, a Ca^2+^ -dependent cysteine protease that induces caspase-12 localization on the cytoplasmic side of the ER^16^,^17^.

In addition, it has previously been reported that in response to a death stimulus such as oxidative stress or DNA damage, a fraction of p53 rapidly translocates to the mitochondria^18^, and this translocation is sufficient to trigger apoptosis. Our preliminary data have indicated that ROS is involved in the signaling events that lead to the mitochondrial migration of p53. Oxidative stress is also thought to play a pivotal role in heat stress induced apoptosis^19^. In fact, we have previously demonstrated that during heat stress induced apoptosis, mitochondrial translocation of p53 is involved in triggering ROS-dependent apoptosis^12^. However, the precise mechanism by which heat stress leads to apoptosis remains largely unclear. In this study, we investigated the potential molecular mechanism that could explain the cellular apoptosis observed in response to oxidative stress caused by the exposure of cells to heat stress. In brief, we focused on two intracellular events, 1) heat stress induced p53 translocation to mitochondria, which in turn induces the mitochondrial apoptotic pathways^12^ and 2) heat stress induced calcium mobilization, mitochondrial calcium overload and the subsequent mitochondria dependent cell death cascade in HUVEC cells. We further elucidated the role of Ca^2+^ dyshomeostasis in the opening of the permeability transition pore (MPTP). In addition, we demonstrated that Bax conformational change and mitochondrial translocation are the key events that link heat stress induced apoptosis to the increased ROS that ultimately acts as an upstream signal triggering the mitochondrial apoptotic pathways.

## Results

### Cell viability and cytotoxicity

To examine the cytotoxic effect of heat stress on HUVEC cells, cells were cultured, harvested at 0, 1, 3, 6 and 9 h after 2 h of heat stress, and then cell viability was quantified using both LDH and WST-1 assays. Cells cultured at 37 °C were used as control. Cell viability was found to have declined drastically and plasma membrane integrity was damaged significantly in a time-dependent manner after exposure to heat stress (supplementary Fig 1). These results suggest that heat stress exerts a cytotoxic effect on HUVEC cells.

### Bax conformational change and mitochondrial translocation promote the mitochondrial apoptotic pathways

**Figure 1:**
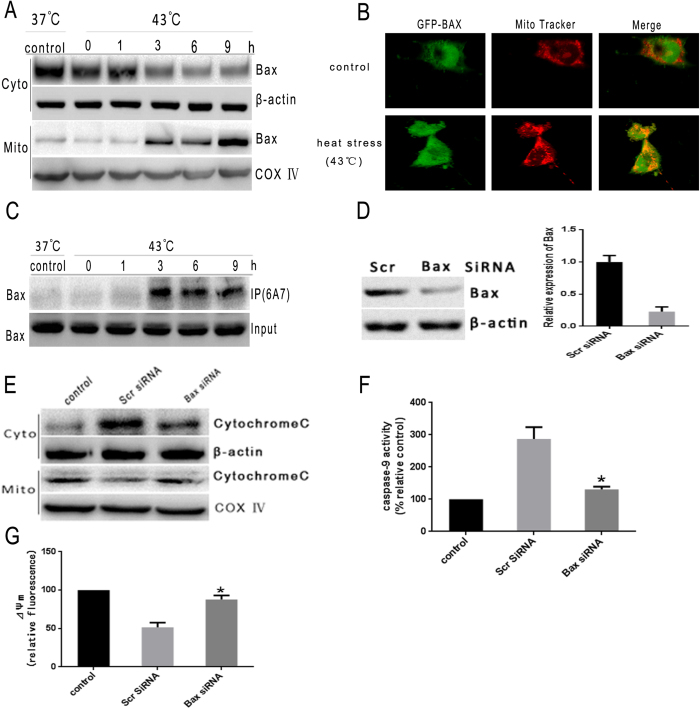
Heat stress induces Bax conformational change and mitochondrial translocation. (**A**) Heat stress induces Bax translocation from the cytosol to mitochondria. HUVEC cells were treated at 43 °C for 2 h, and then further incubated for different durations as indicated, Bax protein levels were assessed by western blot(cropped). COX IV was used as mitochondrial loading control. (**B**) GFP-Bax colocalizes with MitoTracker upon heat stress treatment under confocal microscopy. HUVEC cells were treated with 43 °C for 2 h, cells were further incubated for 6 h. GFP-Bax (green). MitoTracker (red). Merged images are shown in the right panels. (**C**) Heat stress induces Bax conformational change. After HUVEC cells were exposed to heat stress, whole cell lysates were used for immunoprecipitation with conformation-specific Bax (6A7) monoclonal antibody, and the immunoprecipitated complexes were analyzed by western blot(cropped) using a polyclonal Bax antibody. (**D**–**G**) HUVEC transfected cells were treated with 43 °C for 2 h, the cells were further incubated for 6 h, and (**D**) HUVEC cells were transfected with scrambled siRNA (Scr) or Bax siRNA. Bax protein expression and mRNA levels were determined by western blot(cropped) and RT-PCR, respectively. β-actin was used as an internal control. (**E**) Cytochrome c release from the mitochondria is inhibited by knocking down of Bax in heat stress exposed cells. The intracellular location of cytochrome c was determined by western blots(cropped). COX IV was used as mitochondrial loading control. (**F**) Bax knockdown diminishes the loss of *ΔΨm* in heat stress exposed cells. The loss of *ΔΨm* was measured by JC-1 and flow cytometry. (**G**) Caspase-9 activity is reduced in Bax knockdown heat stress treated cells. Enzymatic activity of caspase-9 was measured in cell lysates using fluorogenic substrate Ac-LEHD-AFC and was expressed relative to control incubated at 37 °C (100%). Each value is the mean ± SD of three separate experiments. *P < 0.05 versus scrambled siRNA-transfected cells exposed to heat stress.

We have previously reported that heat stress could induce caspase-9 activation, loss of *ΔΨm* and cytochrome c release, but not caspase-8 or caspase-4 activation. These data indicated that the mitochondrial pathway may mediate the intense heat stress-induced early wave of apoptosis of HUVEC cells^12^. Bax conformational change, accompanied by translocation to the mitochondria, is believed to be a key step that prompts the release of apoptogenic factor from the mitochondria to the cytosol. These factors include apoptosis-inducing factors, cytochrome c and Smac^20^,^21^. Several studies have correlated Bax mitochondrial translocation with the early events that contribute to the release of cytocrhome c from the mitochondria, which is followed by caspase activation in intrinsic apoptosis signaling induced by apoptotic stimuli^22^,^23^. Bax redistribution to the mitochondria was also observed in CHO and HeLa cells exposed to heat stress^24^. To investigate whether heat stress induces Bax mitochondrial redistribution, subcellular fractionation was performed to examine the Bax levels in both cytosolic and mitochondrial compartments. Although total Bax levels remained constant, exposure of HUVEC cells to heat stress for 2 h resulted in a significant increase of Bax in the mitochondria accompanied by the depletion of cytosolic Bax. (Fig. 1A and supplementary Fig. 2). To visualize Bax localization at the mitochondria of early apoptotic cells, we established a GFP-Bax HUVEC cell line that stably expresses human Bax with GFP fused to its N terminus. GFP-Bax fluorescence was found to overlap with Mitotracker staining in heat-treated cells (Fig. 1B). Since conformational change of Bax represents a crucial step in its mitochondria translocation and the subsequent initiation of apoptosis, we utilized a monoclonal antibody that discriminatively recognizes the Bax protein (6A7) with conformational alterations to identify whether heat stress induces conformational changes in Bax protein^25^. We found that exposure to heat stress resulted in an obvious increase in the amount of Bax that had undergone conformational change (Fig. 1C).

To investigate the functionality of Bax in heat stress mediated apoptosis, we examined the effects of Bax knockdown with siRNA on the cellular features indicative of apoptosis. We found that cytochrome c release and caspase-9 activation were reduced in cells containing lower levels of Bax, suggesting that decrease in Bax level may protect cells from heat stress induced apoptosis (Fig. 1D–F). Take together, these findings suggest that Bax, especially its conformational change and mitochondrial translocation, plays a vital role in heat stress induced apoptosis in HUVEC cells.

### Mitochondrial translocation of p53 is involved in Bax conformational change and mitochondrial translocation

**Figure 2:**
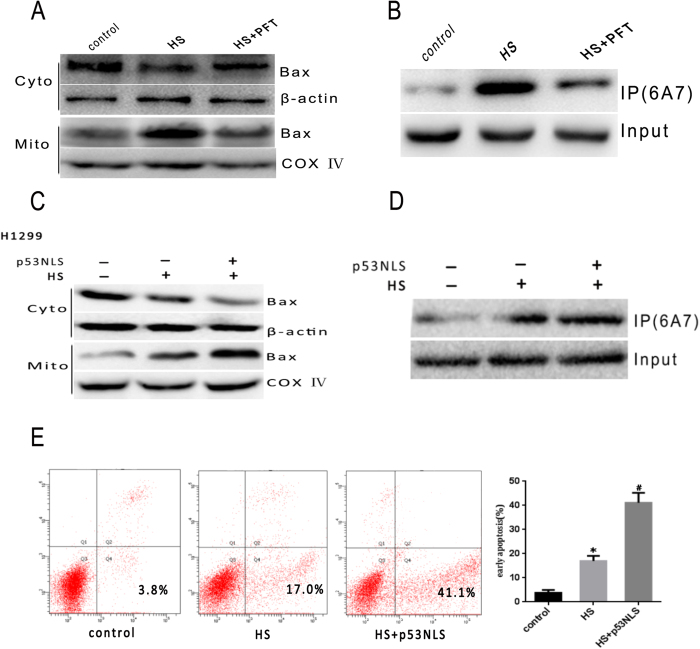
Non-transcriptional p53 is involved in Bax mediated apoptosis. (**A**) Bax mitochondrial translocation in heat stress treated cells is inhibited by PFT inhibition of p53. Bax protein levels in cytosol and mitochondria were analyzed using western blot(cropped). COX IV, mitochondrial loading control. (**B**) Bax conformational change is diminished by PFT inhibition of p53. Bax (6A7) with conformational changes was specifically detected by immunoprecipitation(cropped) with an anti-Bax 6A7 monoclonal antibody. (**C**–**E**) Non-transcriptional p53 apoptosis analysis in H1299 cells overexpressing p53NLS. (**C**) Bax protein levels in cytosol and mitochondria were analyzed using western blot(cropped). COX IV, mitochondrial loading control. (**D**) Bax conformational change is diminished by PFT inhibition of p53. Bax (6A7) with conformational changes was specifically detected by immunoprecipitation(cropped) with an anti-Bax 6A7 monoclonal antibody. (**E**) apoptosis was analyzed by flow cytometry using an Annexin V-FITC/PI staining kit. Data are presented as means ± SD of three separate experiments. *p < 0.05 as compared to control; ^#^p < 0.05 as compared to heat stress (HS) alone.

Results from our previous study have demonstrated that heat stress induces p53 translocation to the mitochondria, which in turn triggers the mitochondrial apoptotic pathways^12^. However, the mechanism by which mitochondrial p53 activates the apoptotic pathways is largely unclear. Other studies have suggested that p53 can directly activate Bax and thereby induce apoptosis^26^,^27^. To confirm activation of Bax by non-transcriptional p53 in heat stress induced apoptosis, we first administered pifithrin-α (PFT), a highly selective and potent synthetic p53 inhibitor, to HUVEC cells to investigate whether PFT caused inhibition of p53 mitochondrial translocation affects Bax^28^. Expectedly, PFT administration significantly reduced Bax conformational change and mitochondrial translocation in the cells exposed to heat stress (Fig. 2A,B). To further verify whether mitochondrial translocation of p53 is a major step in heat stress induced apoptosis, we employed a nuclear import-deficient p53 construct (p53NLS) in p53-deficient cancer cells(H1299). As shown in Fig. 2C–E, p53NLS could efficiently induce apoptosis. These results confirmed that p53 accumulates in mitochondria and activates Bax mitochondrial translocation in response to heat stress.

### Effect of heat stress on Ca^2+^ homeostasis

**Figure 3:**
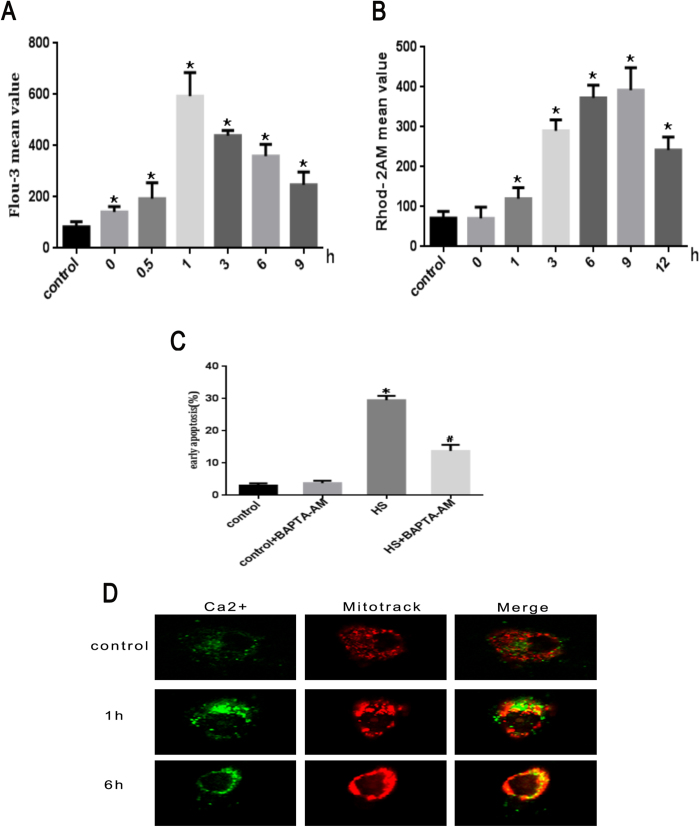
Heat stress induces imbalances of calcium homeostasis in HUVEC cells. (**A**) Cytoplasmic Ca^2+^ level was determined by flow cytometry using the fluorescent probe Fluo-3/AM. (**B**) Mitochondrial Ca^2+^ level was determined by flow cytometry using the fluorescent probe Rhod-2 AM. (**C**) Apoptosis was analyzed by flow cytometry using Annexin V-FITC/PI staining. (**D**) Distribution of Ca^2+^ in cell was analyzed by laser scanning confocal microscopy. Data are presented as means ± SD of three separate experiments. *p < 0.05 as compared to control; ^#^p < 0.05 as compared to heat stress (HS) alone.

Since Ca^2+^ has been shown to play an important role in apoptosis in different cell types^29^, we explored whether heat stress induced apoptosis is associated with calcium imbalance. HUVEC cells were stained with fluorescent probe dihydrorhod-2 AM (Rhod-2 AM) for mitochondrial calcium and Fluo-3-AM for cytoplasmic calcium. Using these dyes, the calcium localization was examined by flow cytometry. Treatment with heat stress led to an initial increase in cytoplasmic Ca^2+^ at 0 h that peaked at 1 h after heat stress and then decreased gradually (Fig. 3A). There was an initial increase in mitochondrial Ca^2+^ at 1 h, and peaked at 9 h after heat stress, then decreased gradually (Fig. 3B). The level of intracellular free Ca^2+^ was the lowest in control group. Ca^2+^ distribution in control cells as well as cells exposed to heat stress for 1 h and 6 h was further analyzed by laser scanning confocal microscopy. We found that Ca^2+^ staining (green) and MitoTracker pattern (red) overlaped at 6 h after subjecting to heat stress (Fig. 3D). These results suggest that heat stress induced an increase in cytoplasmic Ca^2+^, which stimulated mitochondrial intake of Ca^2+^, and potentially led to mitochondrial Ca^2+^ overload.

### Heat stress mediated Ca^2+^ -dependent cell apoptosis via activation of MPTP

**Figure 4:**
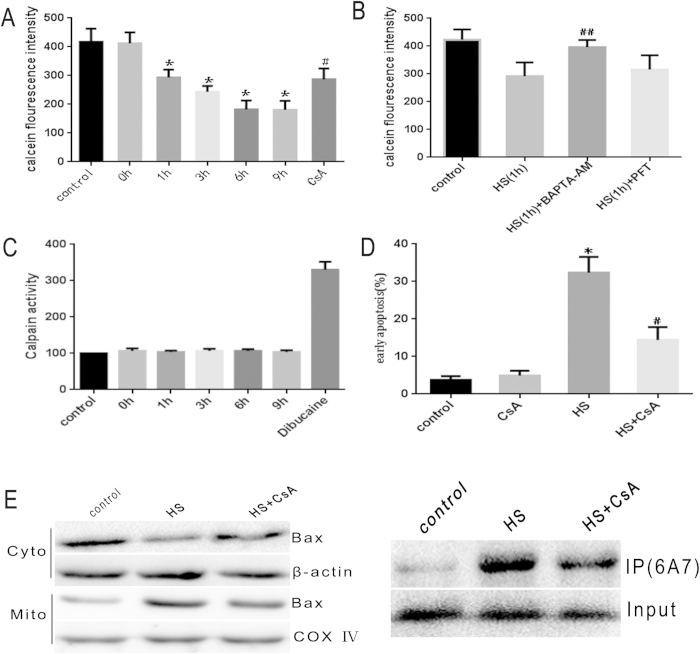
MPTP opening is involved in Bax mediated apoptosis. (**A**) Heat stress induces MPTP opening as evidenced by the gradual decrease in cytosolic calcein. Heat stress treated HUVEC cells were stained with the calcein-AM and COCl_2_ at equal volume mixture, and analyzed for fluorescence intensity using LSCM. (**B**) MPTP opening induced by heat stress is not affected by PFT inhibition of p53. Cells were pretreated with 20 μM BAPTA-AM and PFT, respectively, and then incubated at 43 °C for 2 h, and then further incubated for 1 h. The MPTP opening was measured by staining with calcein-AM and COCl_2_. (**C**) Calpain activitiy is not affected in heat stress induced HUVEC cells. Calpain activities were measured by analyzing the fluorometric cleavage of their substrates Ac-LLY-AFC. HUVEC cells treated with 250 μM Dibucainewere as a positive control. (**D**,**E**) Cells were pretreated with 50 μM CsA, then incubated at 43 °C for 2 h, followed by further incubation for 6 h, and (**D**) Inhibition of MPTP opening diminishes the percentage of early apoptosis in heat treated HUVEC cells. Apoptosis was analyzed by flow cytometry using Annexin V-FITC/PI. (**E**) Inhibition of MPTP opening reduces heat stressed induced Bax conformational change and mitochondrial translocation. Bax mitochondrial translocation was measured by western blot. Bax conformational change was assessed by immunoprecipitation. Data are presented as means ± SD of three separate experiments. *p < 0.05 as compared to control; ^#^p < 0.05 as compared to heat stress (6 h). ^##^p < 0.05 as compared to heat stress (1 h).

Since MPTP has been recognized as one of the major target of Ca^2+^, and opening of MPTP marks the irreversible point of the cell death process^30^, to examine whether calcium-dependent apoptosis induced by heat stress is mediated through MPTP, we stained HUVEC cells for calcein/Co^2+^ to test opening of MPTP. We found the calcein fluorescence intensity in the cytoplasm decreased gradually in a time-dependent manner starting at 1 h after heat stress treatment (Fig. 4A). Pretreatment with BAPTA-AM, an intracellular Ca^2+^ chelator, significantly inhibited MPTP opening at 1 h after heat stress while treatment with PFT did not (Fig. 4B). Although calpain is another major target of Ca^2+^, changes in calpain activity were not found in our experiments. Dibucaine –treated HUVEC cells were used as a positive control^31^ (Fig. 4C). Therefore, the imbalance of Ca^2+^ homeostasis activated MPTP opening without affecting calpain activity in this study. To verify whether MPTP opening is indeed associated with heat stress induced cells apoptosis, HUVEC cells were pretreated with CsA (50 μM), a MPTP inhibitor, for 30 min, followed by exposure to heat stress (43 °C, 2 h). CsA treatment reduced apoptosis from 32.4% to 14.4% (Fig. 4D). In addition, Bax conformational change and mitochondrial translocation were significantly inhibited after pretreatment with CsA (Fig. 4E). These results indicated that MPTP opening is involved in triggering Bax conformational change and mitochondrial translocation in heat stress induced apoptosis.

### Heat stress increases ROS generation in HUVEC cells

**Figure 5:**
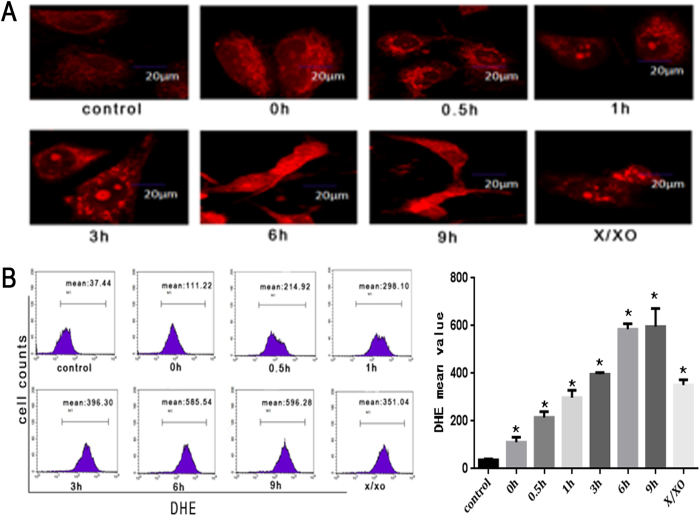
Heat stress induces the production of ROS in HUVEC cells. (**A**) Heat stress treatment leads to increase in ROS generation in HUVEC cells. Laser scanning confocal microscope images of cells stained with DHE were shown (red). The images shown are representative of the three independent image acquisitions. (**B**) Quantitative analysis of the increase in ROS generation upon heat stress treatment in HUVEC cells. The amounts of ROS were analyzed by flow cytometry upon staining with fluorescent dye DHE. X/XO (0.1 mM X + 0.01 U XO) was used as a positive control. Data are presented as mean ± SD for three independent experiments, *P < 0.05 compared with control group (37 °C).

Calcium homeostasis is closely related to ROS accumulation. Excessive ROS intracellular accumulation may lead to calcium dysregulation. Therefore, we explored the possibility that heat stress induced apoptosis triggers ROS accumulation using cell permeable fluorescent dye DHE, which emits enhanced fluorescence upon reacting with ROS. Hypoxanthine/xanthine oxidase (X/XO) -treated HUVEC cells were used as a positive control. We found that the level of intracellular ROS notably increased shortly after exposure to heat stress (0 h), and continued to rise. Quantitative estimation by flow cytometry indicated that DHE fluorescence intensity increased in a time-dependent manner in HUVEC cells after heat stress treatment (Fig. 5B).

### The role of ROS in heat stress induced apoptosis

**Figure 6:**
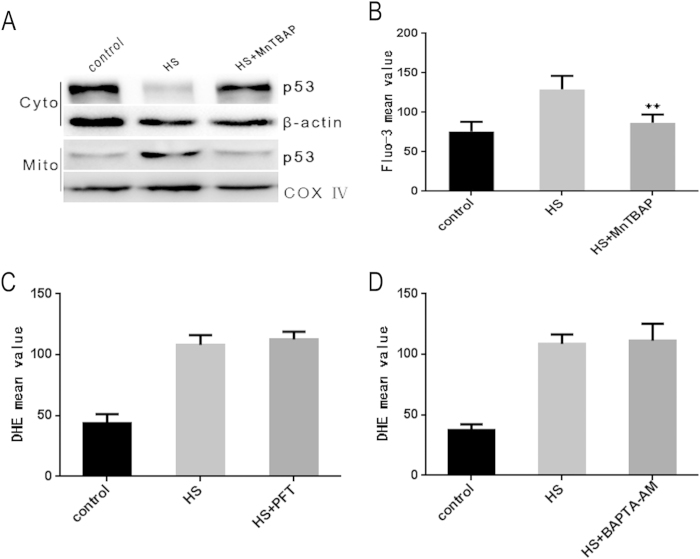
ROS activates mitochondrial translocation of p53 and imbalance of calcium homeostasis in cells exposed to heat stress. (**A**) ROS reduction by MnTBAP pretreatment diminishes mitochondrial translocation of p53 in heat stress exposed HUVEC cells. P53 mitochondrial translocation induced by heat stress was analyzed by western blot. COX IV, mitochondrial loading control. (**B**) ROS reduction by MnTBAP pretreatment decreases heat stress induced Ca^2+^ homeostasis imbalance in heat stress exposed cells. Flow cytometry was used to measure the effect of MnTBAP on Ca^2+^ homeostasis in cells exposed to heat stress. (**C**) PFT inhibition of p53 does not affect heat stress induced ROS generation. ROS production in heat stress exposed cells was measured by flow cytometry after DHE staining. (**D**) Inhibition of MPTP opening does not affect heat stress induced ROS generation. ROS production in heat stress exposed cells was measured by flow cytometry after DHE staining. Data are presented as mean ± SD of three independent experiments. ^**^P < 0.05 compared to HS group (0 h after heat stress).

Our results showed that ROS production, increase in cytoplasmic Ca^2+^ and mitochondrial translocation of p53 occurred at 0 h after exposure to heat stress. To determine the relationship between these three factors, HUVEC cells were pretreated with MnTBAP, BAPTA-AM or PFT, respectively. The cells were subsequently incubated at 43 °C for 2 h, and assessed for DHE and Fluo-3/AM fluorescent intensity by flow cytometry immediately after heat stress (0 h). Localization of p53 was analyzed by western blot. We demonstrated that ROS generation was essentially eliminated upon treatment with the cell permeable ROS scavenger MnTBAP (supplementary Fig. 3). Increase in cytoplasm Ca^2+^ and mitochondria translocation of p53 were also completely inhibited by MnTBAP (Fig. 6A,B). However, the production of ROS was not impacted by BAPTA-AM or PFT (Fig. 6C,D). Overall, our results showed that heat stress induced ROS generation that led to increased concentration of cytoplasm Ca^2+^ and p53 translocation to the mitochondria.

**Figure 7:**
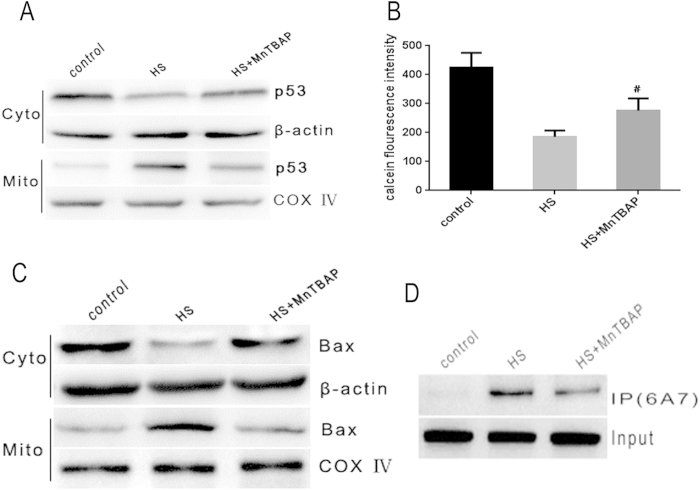
Heat stress induced ROS production is the upstream signal required for the triggering of downstream apoptosis initiating events. (**A**) ROS reduction by MnTBAP pretreatment suppresses p53 mitochondrial translocation in heat stress exposed cells. Protein levels of p53 in cytosol and mitochondria were measured by western blot(cropped). COX IV, mitochondrial loading control. (**B**) ROS reduction by MnTBAP diminishes MPTP opening in heat stress exposed cells. MPTP opening was assessed by staining with calcein-AM and COCl_2_. (**C**,**D**) ROS reduction by MnTBAP diminishes Bax conformational change and mitochondrial translocation in heat stressed exposed cells. Bax tranlocation and conformation change were measured by western blot and immunoprecipitation, respectively.

**Figure 8:**
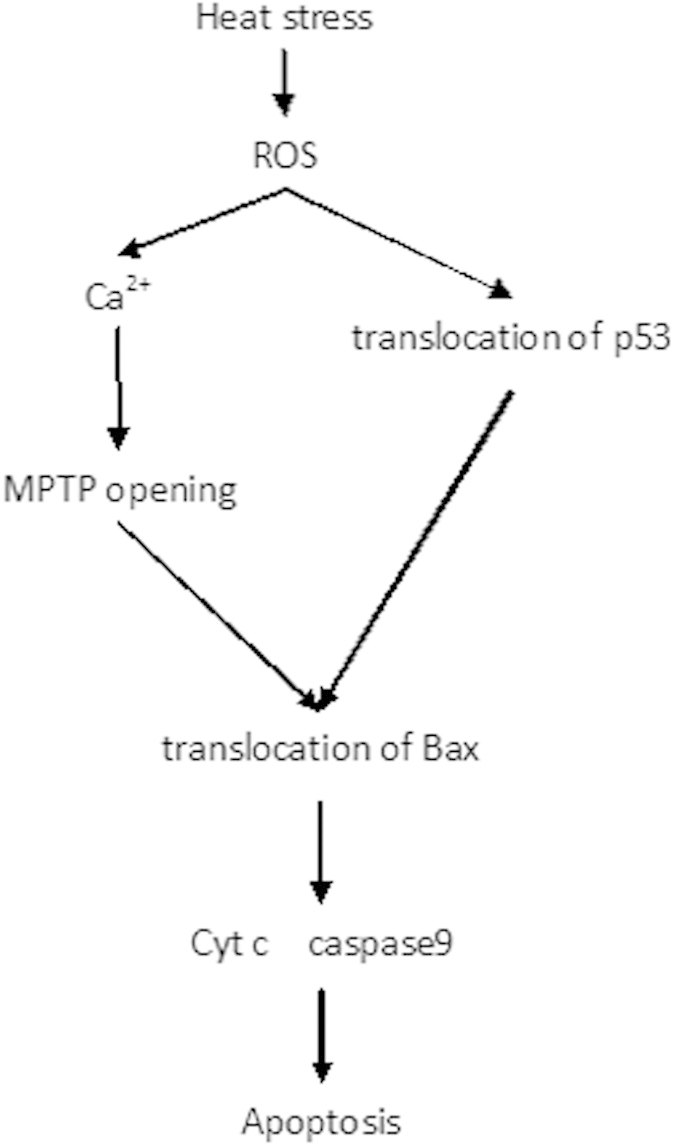
Proposed apoptosis signaling pathways activated by heat stress in HUVEC cells.

To further ascertain the role of ROS in triggering heat stress induced apoptosis signaling pathways, HUVEC cells were pretreated with MnTBAP, followed by an incubation at 43 °C for 2 h, and a further incubation for 6 h. The depletion of ROS by MnTBAP significantly inhibited increase in cytoplasmic Ca^2+^ (supplementary Fig. 4). MnTBAP also significantly inhibited heat stress mediated mitochondrial translocation of p53, MPTP opening and Bax mitochondrial translocation (Fig. 7A–D). Furthermore, decrease in cytochrome c release from the mitochondria, changes in mitochondrial membrane potential and caspase-9 activity were also identified in MnTBAP treated cells (supplementary Fig. 4). These results demonstrated that heat stress induced accumulation of ROS, which then activated transcription-independent p53 and Ca^2+^ -MPTP signaling that mediated mitochondrial apoptotic pathways through inducing conformational change and mitochondrial translocation of Bax (Fig. 8).

## Discussion

In this study, we demonstrated that conformational change and mitochondrial translocation of Bax play a vital role in heat stress induced apoptosis. Heat stress also induces mitochondrial Ca^2+^ overload to activate opening of mitochondrial permeability transition pore (MPTP), which subsequently induces Bax conformational change and mitochondrial translocation that ultimately lead to mitochondrial apoptotic pathway. It is worth noting that both mitochondrial p53 translocation and Ca^2+^ dyshomeostasis are dependent on ROS generation in heat stress induced HUVEC cells apoptosis. To our knowledge, this is the first study that establishes ROS involvement in triggering both mitochondrial p53 translocation and mitochondrial Ca^2+^ overload. Both of these processes contribute to heat stress induced HUVEC cells apoptosis by inducing Bax conformational change and mitochondrial translocation.

The apoptotic process can occur via three different pathways, including death receptors (extrinsic pathway), mitochondria (intrinsic pathway) and endoplasmic reticulum (intrinsic pathway), respectively. There are a series of specific proteins in each of these pathways. The intrinsic pathway leads to activation of caspase-9 and caspase-4/12^32^, while the extrinsic pathway is dependent on caspase-8^33^. In this study, we observed increased caspase-9 and caspase-3 activity in HUVEC cells subjected to heat stress, but caspase-8 and -4 activity was not altered in heat stress exposed cells. In addition, our results also revealed that heat stress leads to a significant increase in cytochrome c released from mitochondria while *ΔΨm* level is diminished. Taken together, our results suggest that heat stress induced apoptosis is likely dependent on the mitochondrial pathway, without involving death receptors or endoplasmic reticulum (ER). Our observations are consistent with other reports, such as report from Hsu YL *et al*. which found that heat stress triggers mitochondrial apoptotic pathway resulting in increased caspase-9 activity^11^, and report from Milleron RS *et al*. which showed that cells undergoing early phase of apoptosis (within 4 h of heat stress exposure) trigger mitochondrial apoptotic pathway by activating apical protease that induces the loss of *ΔΨm* and triggers caspase-3 activation^34^.

Bax is a proapoptotic member of the Bcl-2 family proteins, and it plays an important role in the mitochondrial apoptosis pathway^35^. In healthy living cells, Bax was found to reside predominantly in the cytosol, and migrate to the membrane of mitochondria during apoptosis^35^. This translocation process involves a conformational change in Bax that results in exposure of its C terminal hydrophobic domain^36^. The link between the mitochondrial translocation of Bax and the release of cytochrome c as well as the subsequent induction of apoptosis is well established^37^,^38^. Our results support this model in that suppression of Bax expression by siRNA transfection decreases cytochrome c release and the subsequent heat stress induced apoptosis. Study by BETTAIEB^24^ also suggest that heat stress induced translocation of Bax from cytosol to mitochondria triggers the release of cytochrome c from the mitochondria^24^. Although these studies define Bax translocation as an important component of apoptosis, little is known about the precise underlying mechanism that regulates Bax translocation in response to heat stress. We previously reported that heat stress could activate p53 translocation to mitochondria, and trigger the mitochondrial apoptotic pathways^12^. As one of the p53 downstream target genes, Bax has been shown to be required for p53-dependent apoptosis in some systems^39^. There are other reports showed that p53 is capable of directly activating Bax in the absence of active transcription^39^,^40^. In this 2study, we further connected p53 to Bax by showing that transcription-independent p53 mitochondrial translocation induces conformational change and mitochondrial translocation of Bax, which then triggers the mitochondrial apoptotic pathway.

Ca^2+^ is often used by cells as a second messenger that translates extracellular stimuli into intracellular activities that are important for regulation of cell survival, development, gene expression and differentiation^40^. Ca^2+^ dyshomeostasis has been implicated as a pivotal mediator in the effector phase in various stress induced cell apoptosis. It is also well established that mitochondrial Ca^2+^ overload is a common feature in cells suffering from various death stressors^41^,^42^. In this study, we demonstrated that heat stress began to increase the intracellular calcium concentration in heat-treated cells at 0 h after exposure, followed by a decrease in cytosolic calcium, along with an increase in mitochondrial Ca^2+^ increase in cells exposed to heat stress for up to 6 h (Fig. 7). Mitochondrial Ca^2+^ overload can lead to activation of its downstream targets, calcineurin and calpains, or the prolonged opening of MPTP. These events can cause pathological consequences, such as apoptosis^43^,^44^. Indeed, we found that heat stress led to MPTP opening, but not activation of calpains. Once the MPTP is opened, various molecules could enter mitochondria non-selectively, which could result in mitochondrial depolarization and oxidative phosphorylation uncoupling. Other research also indicated that the opening of MPTP could lead to a decrease in mitochondrial membrane potential, cytochrome c release, and eventrally the induction of apoptosis^15^. In this study, we demonstrated that MPTP opening is involved in signaling for Bax translocation to the mitochondria. It remains unclear how MPTP opening activates Bax translocation to the mitochondria, however, MPTP opening has been shown to lead to changes in mitochondrial shape^45^, which could potentially contribute to Bax migration in the presence of dynein.

Several reports have linked oxidative stress with heat stress and suggested a synergistic augmentation of cell death as increased ROS generation was observed in heat-exposed cells^46^,^47^,^48^. Furthermore, recent studies have revealed that ROS plays a dual role by acting as an upstream signal that triggers p53 translocation and Ca^2+^ dyshomeostasis, while also acts as a downstream factor that mediates apoptosis^49^,^50^. Our findings indicate that heat stress increases ROS generation, along with mitochondrial p53 translocation and Ca^2+^ accumulation in HUVEC cells. Using MnTBAP, we demonstrated that ROS plays a vital role in activating heat stress induced apoptosis.

In conclusion, our study provides the first demonstration of the potential mechanism by which heat stress exposure leads to apoptotic cell death. Our findings indicated that heat stress affects multiple processes, such as mitochondrial translocation of transcriptional-independent p53 and the calcium-mediated MPTP opening. These two processes then induce conformational change and mitochondrial translocation of Bax, which in turn, activates subsequent caspase cascade. Our results also showed that ROS is as an upstream signal molecule involved in heat stress-induced HUVEC cells apoptosis.

## Materials and methods

### Cell culture, treatments and cell viability assays

Human umbilical vein endothelial cells (HUVECs) were purchased from Shanghai Institute of Cell Biology, Chinese Academy of Sciences. Cells were grown in media recommended by the manufacturer. Culture dishes containing HUVEC cells in media were sealed with parafilm and were immersed in a thermo-regulated circulating water bath at 37 °C ± 0.5 °C for 2 h for the control treatment or at 39 °C, 41 °C, 43 °C, or 45 °C ± 0.5 °C for the corresponding heat stress treatments^12^,^51^. Culture media were replaced with fresh media and the fed cells were further incubated for the respective indicated time. Cell viability was assessed by Premixed WST-1 Cell Proliferation Reagent (Clontech Laboratories Inc., Mountain View, CA, USA) according to the manufacturer’s instructions. The enzyme activity of Lactate dehydrogenase (LDH) was assayed using a commercial kit (JianChen Co, Nanjing, China) according to the manufacturer’s instruction.

### Flow cytometry analysis of cell apoptosis using Annexin V-FITC/PI staining

Cells were either kept untreated or exposed to 43 °C for 2 h before analysis by flow cytometry. The detection was performed according to the manual of Annexin V-FITC apoptosis detection kit (invitrogen). About 1 × 10^6^ cells were collected, washed with ice-cold PBS, and resuspended in binding buffer containing suitable amount of Annexin V-FITC. After 10 min of incubation in the dark at room temperature, the buffer was removed by centrifugation. The cells were then resuspended in reaction buffer containing propidium iodide (PI). Flow cytometry analysis was performed immediately to detect apoptosis.

### Assay for caspase activity

After treatment at 43 °C for 2 h, cells were harvested, and cell lysates were prepared at −80 °C for 30 min and then incubated at 37 °C with the following appropriate caspase substrates using a Quadruple Monochromator Microplate Reader (Infinite M1000, Tecan US, NC, USA). Caspase activities were measured by cleavage of the following fluorogenic peptide substrates^52^: Ac-LEHD-AFC for caspase-9, Ac-DEVD-AMC for caspase-3, Ac-ATAD-AFC for caspase-8 and Ac-LEVD-AFC for caspase-4. Caspase activities are represented as relative cumulative fluorescence of the kinetic reaction as compared to that of untreated controls.

### Enzymatic Assay for Calpain Activity

Calpain activity was tested using Ac-LLY-AFC as the substrate provided in the calpain activity fluorometric assay kit (BioVision). Cells (1 × 10^6^) were resuspended in 100 μl of extraction buffer and centrifuged at 10000 g for 1 min. Then 100 μg cell lysate was dilute in 85 μl extraction buffer, and the cell lysate was transferred to a 96-well plate to which 10 μl reaction buffer and 5 μl calpain substrate were added to each well. After incubation at 37 °C for 1 h in the dark, the absorbance was measure using a fluorometer equipped with a 400 nm excitation filter and a 505 nm emission filter. 250 μM Dibucaine –treated HUVEC cell sample was used as a positive control.

### Measurement of cytoplasmic and mitochondrial calcium

To follow the heat stress-induced cytoplasmic and mitochondrial calcium trafficking, cytosolic and mitochondrial Ca^2+^ was estimated by co-incubating the cells with a cell permeant but mitochondria impermeant Ca^2+^ fluorophore, Fluo-3 AM (2 μM) and mitochondria-permeant Ca^2+^ fluorophore, Rhod-2 AM (2 μM), respectively. The changes of cytoplasmic and mitochondrial calcium were evaluated by flow cytometry.

### Measurements of ROS

To analyze the kinetics of ROS generation in HUVEC cells under heat stress. Cells were treated at 43 °C for 2 h, and further incubated for 0, 0.5, 1, 3, 6 and 9 h, respectively. ROS were detected using the fluorescent probe dihydroethidium (DHE, Molecular Probes, Beyotime). HUVEC cells were incubated with 2 μM DHE at 37 °C in the dark for 30 min. The fluorescence intensity was measured by flow cytometry and the cellular images were captured using laser scanning confocal microscope.

### Calcein/Co^2+^ assay for assessing mitochondrial permeability transition pore (PTP) opening

Calcein-AM is a non-fluorescent, cell permeable and hydrophilic compound that is widely used for detection of cell viability. In live cells, the hydrolysis of calcein-AM by intracellular esterases produces strongly green fluorescent calcein, a hydrophilic compound that is well retained in the cell cytoplasm. Mitochondria were stained with the acetomethoxy derivate of calcein (calcein–AM) through Co^2+^ quenching of cytosolic calcein florescence. This nature of calcein-AM allows for the assessment of MPTP opening. HUVEC cells were cultured and treated at 43 °C for 2 h, followed by an incubation with 1 μM calcein-AM for 15 min, then the medium was changed to calcein-free medium containing 1 μM CoCl_2_ and incubated at 37 °C for 20 min in the dark. Then the cells were washed with ice-cold PBS (pH 7.2) for four times and the fluorescence intensity of cells exposed to heat stress or untreated control cells was measured with excitation at 490 nm and emission at 520 nm.

### Mitochondrial membrane potential assay

The mitochondrial membrane potential was detected using 5,5′, 6,6′-tetrachloro-1, 1′, 3,3′ tetraethylbenzimidazolcarbocyanine iodide (JC-1; Molecular Probes, Eugene, OR, USA). Following heat stress treatment, cells were rinsed once with complete culture medium and incubated with JC-1 at 37 °C for 30 min. Prior to fluorescence detection, cells were centrifuged, washed twice with cold PBS, and transferred to a 96-well plate (10^5^ cells/well). The plate was then analyzed with a fluorescence plate reader under the following settings: excitation at 490 nm, emission at 540 nm and 590 nm. Changes in the ratio between the fluorescence intensity measurement at 590 nm (red) and 540 nm (green) are indicative of changes in the mitochondrial membrane potential^53^.

### Preparation of subcellular fractions

HUVEC cells were pretreated at 43 °C for 2 h and then incubated for different durations. Untreated cells were used as control. Subcellular fractions were separated as previously described^54^,^55^. Cell lysates were homogenised using a dounce homogeniser (50 strokes/sample). After an incubation on ice for 30 min, the unbroken cells and nuclei were pelleted by centrifugation at 2500 g for 10 min. The supernatant was collected and further centrifuged at 13,000 g for 15 min. The pellet containing the mitochondrial fraction was then resuspended in buffer C (300 mM sucrose, 1 mM EGTA, 20 mM MOPS, 0.1 mM DTT and 100 l l/10 ml of cocktail of protease inhibitors, pH 7.4). P53 and cytochrome c levels in the mitochondrial fraction were then determined. The remaining supernatant was further centrifuged at 100,000 g for 1 h. The final supernatant was designated as the cytosolic fraction, which was used for detection of Bax and cytochrome c.

### Western blot

HUVEC cells were pretreated at 43 °C for 2 h, and then incubated for different durations. Western blot was carried out with monoclonal anti-p53 antibody (1:1000; Cell signaling technology, Danvers, USA), polyclonal anti-Cytochrome C antibody (1:1000; Cell signaling technology, Danvers, USA) and polyclonal anti-Bax antibody (1:200, Santa Cruz) as previously described^56^,^57^. An HRP-conjugated anti-rabbit IgG antibody was used as secondary antibody (Zhongshan Inc, China). Antibody binding signals were detected using enhanced chemiluminescence reagents (Pierce, Rockford, IL, USA).

### Detection of conformational change in Bax

The assay for detecting Bax conformational change was performed as described previously^36^. Briefly, HUVEC cells were lysed with Chaps lysis buffer (10 mM Hepes: pH 7.4, 150 mM NaCl, 1% Chaps and protease inhibitors). Cells protein samples were incubated with anti-Bax 6A7 monoclonal antibody and then with protein G-agarose beads. The beads were washed three times with Chaps buffer, boiled in loading buffer, and then analyzed using western blot.

### Cell Transfection

The pEGFP-C3-Bax constructs were created using a Quick Change site directed mutagenesis kit (Stratagene, La Jolla, CA) according to the manufacturer’s instructions. pcDNA3-p53NLS was generated by subcloning using pCMV BamNeop53NLS^58^. To establish Bax-stable transfected cells or p53NLS-stable transfected cells, HUVEC and H1299 cells were transfected with pEGFP-C3-Bax or p53NLS plasmid. Positive clones overexpressing Bax or p53NLS were selected with 1 mg/ml G418 as described previously^59^. Small-interfering RNA (siRNA) for Bax was designed and synthesized by Guangzhou RiboBio (RiboBio Inc, China), which was transfected into cells using TurboFect^TM^ siRNA Transfection Reagent (Fermentas, Vilnius, Lithuania) according to the manufacturer’s protocol. Cells were collected after 48–72 h for further experiments. The mRNA and protein levels of Bax were estimated by RT-PCR and western blot, respectively.

### Statistical analysis

All of the data were analyzed for statistical significance using SPSS 13.0 software (SPSS, Chicago, IL, USA). Results were expressed as means ± SD from at least 3 independent experiments performed in duplicate. Statistical comparisons of the results were carried out using One-way ANOVA analysis. p < 0.05 was considered statistically significant.

## Additional Information

**How to cite this article**: Gu, Z. T. *et al*. Heat stress induced apoptosis is triggered by transcription-independent p53, Ca2^+^ dyshomeostasis and the subsequent Bax mitochondrial translocation. *Sci. Rep*. **5**, 11497; doi: 10.1038/srep11497 (2015).

## Supplementary Material

Supplementary Information
