# Supplementary material for: Heat stress induced apoptosis is triggered by transcription-independent p53, Ca2+ dyshomeostasis and the subsequent Bax mitochondrial translocation
Source: Sci Rep. 2015 Jun 24;5:11497. doi: 10.1038/srep11497 (PMC4478470; doi:10.1038/srep11497)
Supplement: Supplementary Information [file srep11497-s1.pdf]

Heat stress induced apoptosis is triggered by transcription-independent p53, Ca^{2+} dyshomeostasis and the subsequent Bax mitochondrial translocation

ZT Gu*[1,6], L Li[1,6], F WU[2,6] P Zhao[3,6] H Yang[1], YS Liu[4], Y Geng[4], M Zhao*[5],L Su*[4]

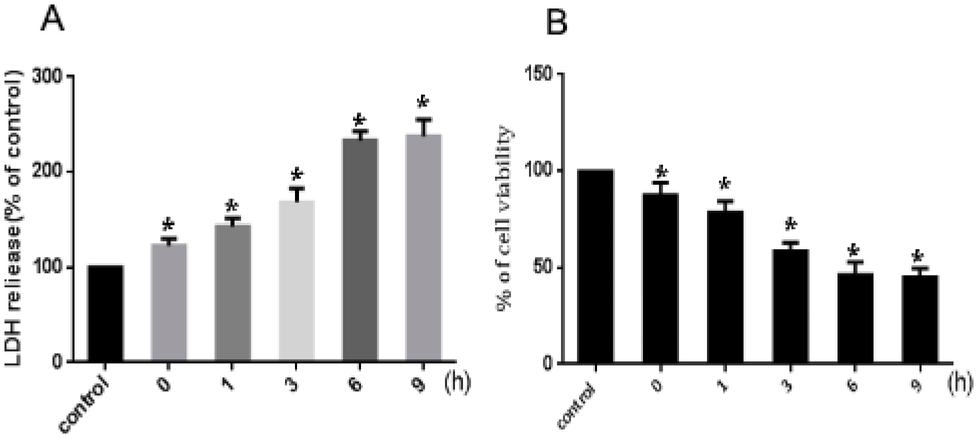

Supplementary Figure1 Exposure to 43℃ for 2h gradually induces cell damage and cell death. (A) Heat stress exposure contributes to increase cell damage as indicated by LDH release. (B) Heat stress treatment reduces cell viability. Each value is the mean ± SD of three independent experiments. *p < 0.05 as compared to control.

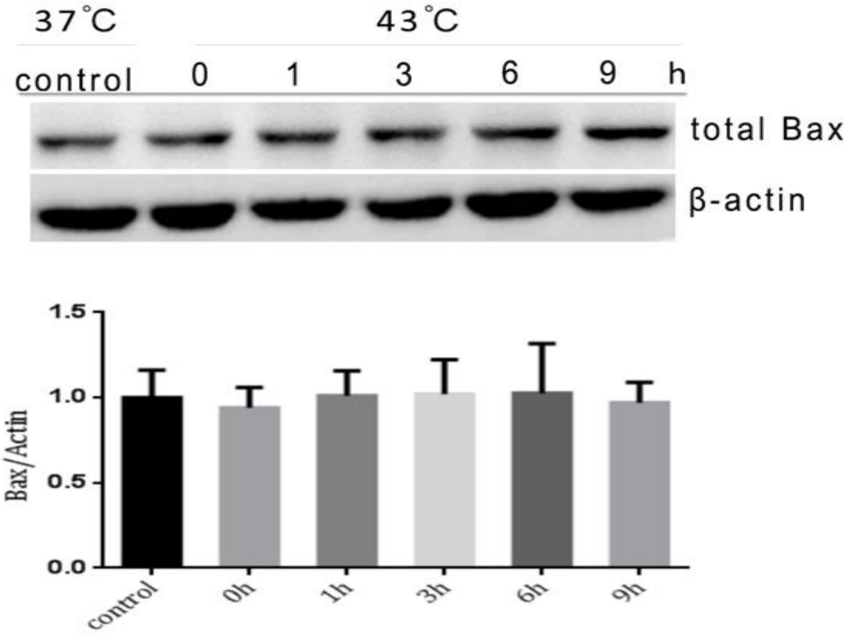

Supplementary Figure 2 Total Bax protein levels is not affected by heat stress exposure. Total Bax levels were measured by western blot and quantify relative to beta-actin loading control (cropped). Each value is the mean ± SD of three independent experiments., *p < 0.05 as compared to control.

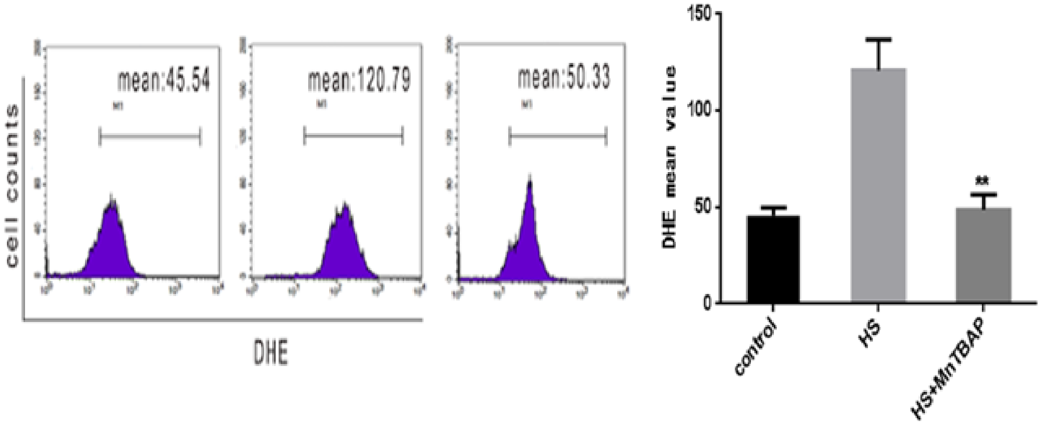

Supplementary Figure 3 Pretreatment with MnTBAP diminishes heat stress induced ROS generation in HUVEC cells. Each value is the mean ± SD of three independent experiments., *$p < 0.05$ as compared to HS control.

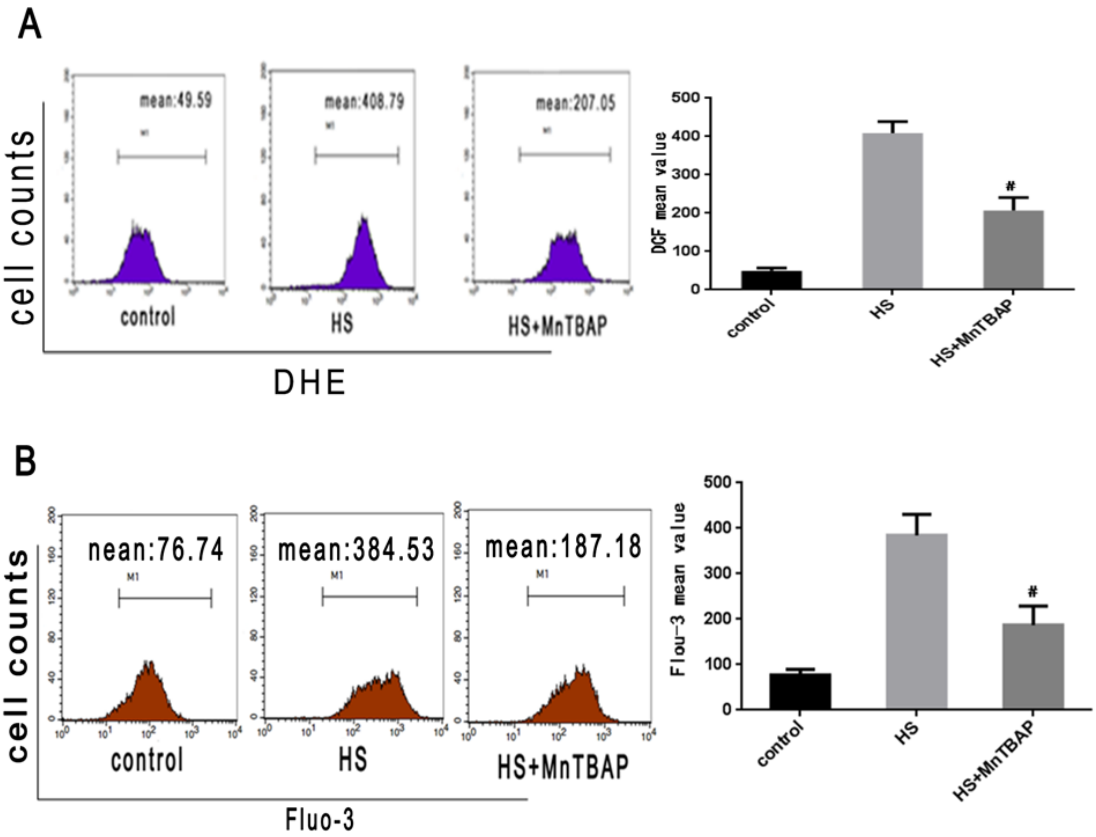

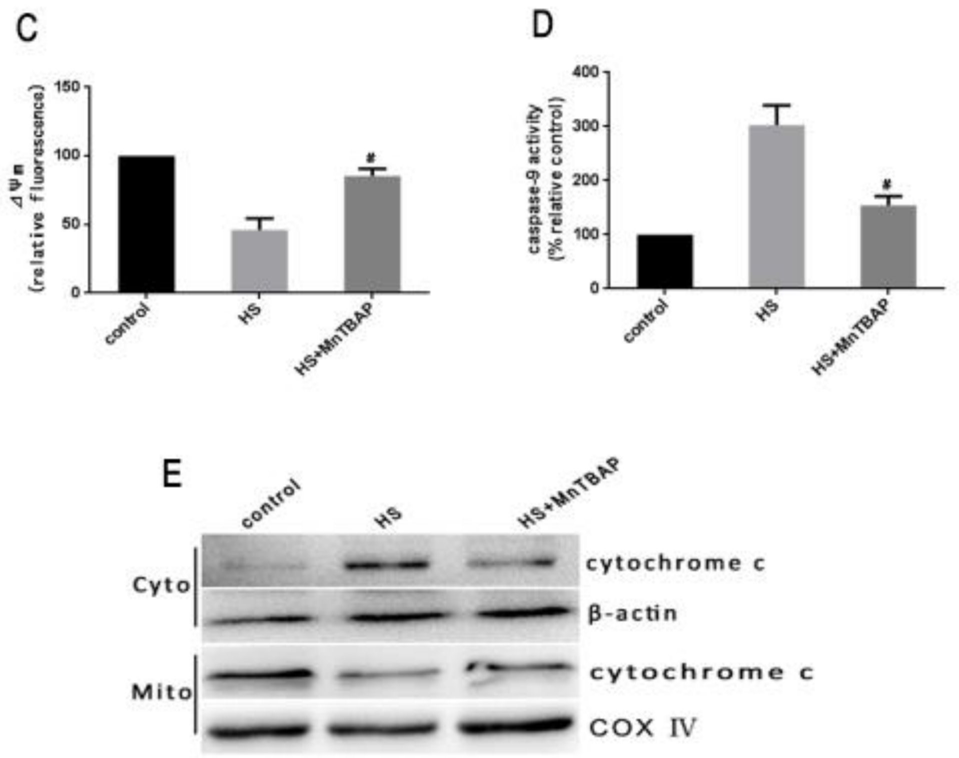

Supplementary Figure 4 Heat stress induced Ca^{2+} dyshomeostasis, cytochrome c release and caspase-9 activity in HUVEC cells are depdent on ROS generation. (A) Pretreatment with MnTBAP effectively diminishes heat stress induced ROS generation in cells used for Ca^{2+} dyshomeostasis analysis. (B) Pretreatment with MnTBAP diminishes heat stress induced Ca^{2+} dyshomeostasis. Flow cytometry of Fluo-3 staining in cells with heat stress. (C) Pretreatment with MnTBAP diminishes the loss of $\varDelta\Psi m$, indicative of reduced cytochrome c release into the cytosol. (D) Pretreatment with MnTBAP inhibits enzymatic activity of caspase-9 measured in cell lysates using fluorogenic substrate Ac-LEHD-AFC. (E) Pretreatment with MnTBAP reduces the amount of cytosolic cytochrome c as determined by western blot (cropped). COX Ⅳ, mitochondrial loading control. Data are presented as mean ± SD of three independent experiments. Each value is the mean ± SD of three independent experiments., *p < 0.05 as compared to control. #P < 0.05 versus HS group (6h after heat stress).
